# Frequency of sabA Gene in Helicobacter pylori Strains Isolated From Patients in Tehran, Iran

**DOI:** 10.5812/ircmj.5044

**Published:** 2013-09-05

**Authors:** Zahra Pakbaz, Mohammad Hasan Shirazi, Reza Ranjbar, Mohammad Reza pourmand, Mohammad Khalifeh Gholi, Amir Aliramezani, Ziba Vaise Malekshahi

**Affiliations:** 1 Department of Pathobiology, School of Public Health, Tehran University of Medical Sciences, Tehran, IR Iran; 2 Molecular Biology Research Center, Baqiyatallah University of Medical Sciences, Tehran, IR Iran; 3 Master of Sciences, Microbial Biotechnology

**Keywords:** SabA protein, Helicobacter pylori, Gene Frequency, Gastric Ulcer

## Abstract

**Background:**

The importance of sialic acid binding adhesin (*sabA*) as a new outer membrane protein in gastroduodenal diseases has been recognized. The prevalence rate of *sabA* gene varies in different geographic areas.

**Objectives:**

The aim of this study was to determine the frequency of *sabA* gene in *Helicobacter pylori* (*H. pylori*) strains isolated from different clinical outcomes in Tehran, Iran.

**Patients and Methods:**

The study included 120 patients with dyspeptic symptoms admitted to the endoscopy suite of gastroenterology section of Firouzgar University Hospital, Tehran, Iran from March to August 2011. Gastric biopsy specimens were evaluated for the presence of *H. pylori* using standard microbiological method and polymerase chain reaction (PCR) assay. The *sabA* genopositive was determined by PCR in *H. pylori* strains.

**Results:**

*H. pylori* isolates were recovered from 82 patients with duodenal ulcer (DU; n = 17), gastric ulcer (GU; n = 15), gastric cancer (GC; n = 13), and gastritis (G; n = 37). The frequency of *sabA* gene in *H. pylori* strains was 100% in gastric cancer, 86.7% in gastric ulcer, and 83.3% in both gastritis and duodenal ulcer.

**Conclusions:**

This is a report on the prevalence of *sabA* gene in *H. pylori* isolated from different gastric patients in Iran. The results showed a high prevalence of *sabA* in our clinical *H. pylori* isolates.

## 1. Background

*Helicobacter pylori* as a gram negative, curved, microaerophilic and motile organism with multiple polar flagella is the most common cause of gastric diseases including gastritis, duodenal and gastric ulcer, gastric atrophy, adenocarcinoma, and mucosa associated lymphoid tissue (MALT) (1). *H. pylori* infection is a major health problem in many parts of the world, occurring in 40 - 50% and 80 - 90% of the population in developed and developing countries respectively (2).

Several studies have shown that host and bacterial factors can play essential role in the appearance of clinical and pathologic complications. Among the bacterial factors, the ability of bacteria to bind to gastric epithelial cells is a crucial step for a successful infection, because it protects the bacteria from cleaning mechanisms such as fluid flow or pouring the mucous layers (3-7).

*H. pylori* has a wide range of adhesion components to bind to different carbohydrates. Sialic acid binding adhesin (*sabA*) plays a critical role in the initial colonization of *H. pylori*, and this molecule has an important role in the establishment of persistent infection and chronic inflammation, which causes tissue damage (8, 9).

*sabA*, a 70-kDa (651-aa protein) outer membrane protein, is essential for attachment and activation of human cells via sialyl-Lewis x/a antigens (sLex and sLea) (10).

## 2. Objectives

The clinical prevalence of the *H. pylorisabA* genotype has not yet been determined in clinical isolates in Iran. In this study, we investigated the presence of *sabA* gene in Iranian clinical *H. pylori* isolates with different clinical outcomes.

## 3. Patients and Methods

### 3.1. Study Population

One hundred twenty patients with dyspeptic symptoms were enrolled in the endoscopy suite of gastroenterology section of Firouzgar University Hospital, Tehran, Iran from March to August 2011. All patients were given informed consent for gastroscopic biopsy samples, which were obtained from the antrum.

### 3.2. Detection of H. pylori and the Presence of sabA Gene

Two gastric biopsy specimens were recovered from patients with gastrointestinal disease. One biopsy was evaluated for rapid urease test (RUT), and the other was sent to laboratory for DNA extraction. DNA was extracted using the DNeasy blood & tissue kit (Qiagen Germany). The sample was considered *H. pylori *positive when a 411bp fragment of urease A gene (ureA) was amplified and RUT made positive. For the *H. pylori *-positive samples, the presence of *sabA *in *H. pylori *clinical isolates was analyzed by PCR using two primer pairs. PCR primers are listed in Table 1. Amplification was performed as previously described ( 11 - 13 ).The PCR products were analyzed by 1% agarose gel electrophoresis with ethidium bromide staining. 

**Table 1.** The Primers used for the Amplification of ureA and*sabA*in This Study

Target gene	Primer Sequence (5–3)	Size of PCR product	Reference
***Urea***	F-CCCAATGGTAAATTAGTT	411bp	(11)
	R-CTCCTTAATTGTTTTTAC		
***sabA*** ** (Pair 1)**	F-CTTTAAGGAACATTTTATGAAAA	785bp	(12, 13)
	R-CACCGCGTATTGCGTTGGGTA		
***sabA*** **(Pair 2)**	F-CCGCTAGTGTCCAGGGTAAC	1330bp	(12)
	R-CGCGCTGTAAGGGTTATTGAAC		

### 3.3. Data analysis

Fisher’s exact test was used for analyzing categorical data. A P-value < 0.05 was considered statistically significant.

## 4. Results

From 120 patients, *H. pylori *isolates were recovered from 82 patients with duodenal ulcer (DU; n = 17), gastric ulcer (GU; n = 15), gastric cancer (GC; n = 13), and gastritis (G; n=37). The mean age of the patients (50 male and 32 female) was 46 years. The presence of *sabA *gene was examined in all 82 of the *H. pylori *-infected patients with gastrointestinal diseases. The strains were considered *sabA *positive if they had one or two fragments of the gene. Two PCR assays yielded different positive rates for the *sabA *gene in 82 *H. pylori *isolates as shown in Table 2.

**Table 2.** Detection of*sabA*Gene by PCR in 82*H. pylori*Clinical Isolates

Primer pair 1	Primer pair 2	Number
**+**	+	60
**_**	+	7
**+**	_	4
**_**	_	11

Seventy one strains (86.6%) had positive results for *sabA *. Table 3 shows the frequency of *sabA *gene among different clinical outcomes. The *sabA *genotype was detected in all patients with gastric cancer (100%), in 86.7% of patients with gastric ulcer, and in 83.3% of patients with gastritis and duodenal ulcer. 

**Table 3.** The Prevalence of*sabA*in the Different Disease Groups

Gene	Clinical outcomes			
	DU	GU	GC	G
**Positive*sabA***	15	13	13	30
**SabA*Negative***	3	2	0	6
**Total**	18	15	13	36

**Figure 1.**
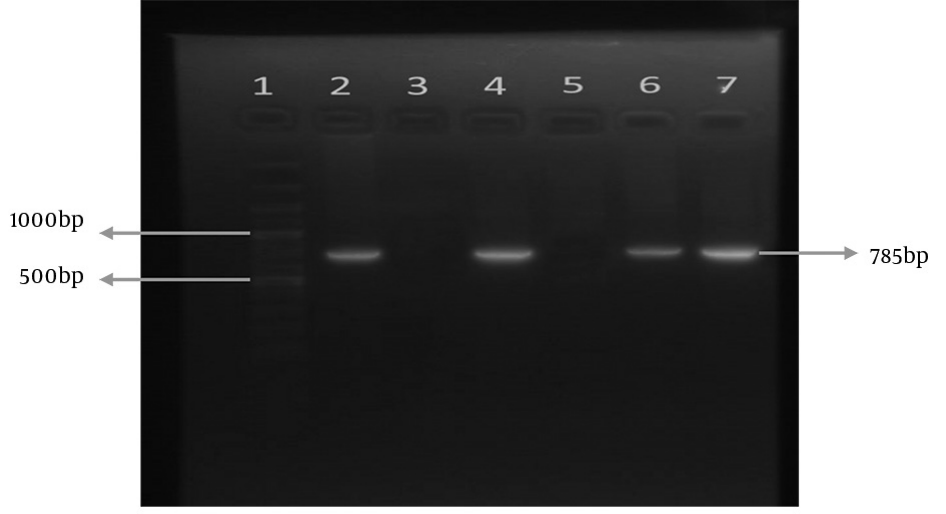
Amplification of sabA Using Primer Pair 1 in Representative Strains of H. pylori. Lane 1 is the 100 bp marker. Lanes 2 and 3 are positive and negative control strains respectively. Lanes 4 to 7 are representative clinical H. pylori strains.

**Figure 2.**
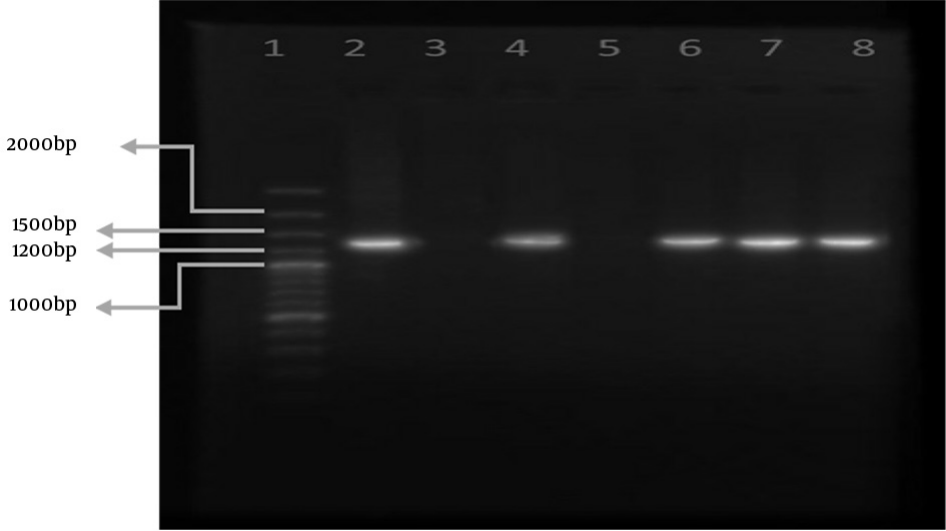
Amplification of sabA Using Primer Pair 2 in Representative Strains of H. pylori. Lane 1 is the 100 bp marker. Lanes 2 and 3 are positive and negative control strains respectively. Lanes 4 to 8 are representative clinical H. pylori strains.

Figure 1 and 2 show the results of *sabA *gene amplification in some strains of *H. pylori *using primers pair 1 and 2 respectively. 

## 5. Discussion

The adherence of *H. pylori* to gastric epithelial cells has a crucial role in the specific tropism and pathogenicity of the organism in the human gastric epithelium and is a key step in creating a successful infection in gastric mucosa (3, 14-16). *sabA* is an important adhesion molecule in *H. pylori* which adheres to the host gastric epithelium (17).

In a previous study performed on 200 *H. pylori*-infected patients, including 120 from Colombia and 80 from the United States, the prevalence rates of *sabA*-positive isolates were 44% in duodenal ulcer, 66% in gastritis, and 70% in gastric cancer (18). In a study in Taiwan, 80% (116 of 145) of *H. pylori* strains had positive results for *sabA* (19). In two other studies the frequency of *sabA*-positive isolates was reported 86% (37 of 43) and 93% (89/96) in French and Dutch respectively (20, 21). Moreover, the prevalence of *sabA* gene was 23.6% in Iran (22).

When compared to previous studies, our results showed that the *sabA* gene has high frequency in Iranian clinical isolates (86.6% (71 of 82)). The frequency of *sabA* in different clinical outcomes was 100% in gastric cancer, 86.7% in gastric ulcer, and 83.3% in both gastritis and duodenal ulcer. However no significant association was seen between the prevalence of *sabA* genotype and clinical outcomes (P > 0.05).

Two PCR assays produced different positive rates for the *sabA* gene in *H. pylori* isolates, with the 81.7% and 78% using primer pairs 1 and 2 respectively; indicating the possibility of sequence diversity in the *sabA* gene (12).

In conclusion, this is a report on the prevalence of *sabA* gene in *H. pylori* isolated from different gastric patients in Iran. More studies in other parts of country with a higher sample size are necessary to be performed to obtain a complete evaluation on the prevalence of *sabA* gene in Iran. Further tests should be performed to determine the exact role of *sabA* in *H. pylori* infection.
